# Subacute Thyroiditis Associated with COVID-19

**DOI:** 10.1155/2020/8891539

**Published:** 2020-09-28

**Authors:** Eugenia Campos-Barrera, Teresa Alvarez-Cisneros, Mario Davalos-Fuentes

**Affiliations:** ^1^Hospital Angeles Del Pedregal, Camino de Sta. Teresa 1055-S, Heroes de Padierna, La Magdalena Contreras, Ciudad de Mexico 10700, Mexico; ^2^Instituto Nacional de Geriatría, Av Contreras 428, San Jeronimo Lidice, Magdalena Contreras, Mexico City 10700, Mexico; ^3^Instituto Nacional de Rehabilitacion, Arenal de Guadalupe, Tlalpan, Mexico City 14389, Mexico

## Abstract

Subacute thyroiditis is a self-limiting inflammatory disorder, characterized by neck pain or discomfort, a tender diffuse goiter, and sometimes a transient episode of hyperthyroidism followed by euthyroidism and sometimes hypothyroidism. There is usually a normalization of thyroid function within a few weeks. Subacute thyroiditis has a higher incidence in summer and has been linked to a viral or bacterial upper respiratory postinfection inflammatory response. We hereby describe the case of a previously healthy 37-year-old female presenting with subacute thyroiditis associated with a very mild presentation of COVID-19. As most patients with SARS-Cov-2 are asymptomatic, we suggest to rule out SARS-Cov-2 infection in patients presenting with symptoms suggesting SAT.

## 1. Case Presentation

On April 10, a previously healthy 37-year-old female experienced odynophagia and anosmia with no other respiratory symptoms. Her laboratory exams confirmed COVID-19 (RT-PCR for SARS-Cov-2) and was prescribed symptomatic treatment for her mild disease improving completely in the following days.

**Figure 1:**
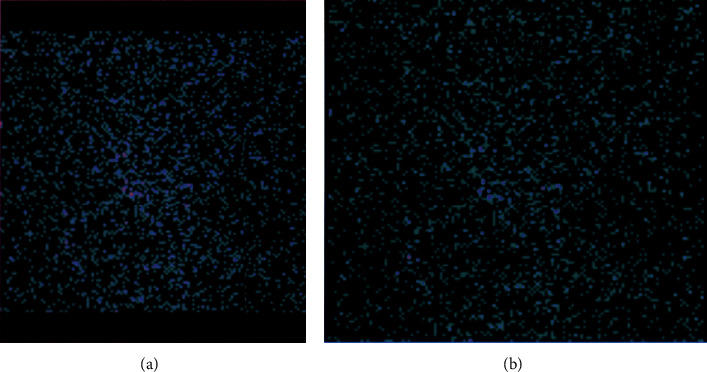
(a, b) The patient's iodine thyroid scan with no uptake by the thyroid gland.

A month after her initial presentation, she presented to the ENT doctor with severe neck pain (8/10) irradiating to the right jaw and ear as well as fatigue. She did not mention any clinical signs of hyperthyroidism (tremor, anxiety, or diaphoresis) but was referred to the endocrinologist with the suspicion of SAT. At this time, her physical exam was significant only for a moderately enlarged tender thyroid gland and neck adenopathies. Her lab test results showed elevated ESR (72 mm/hr) and CRP (66 mg/L), anemia (Hb10.4 g/dL), and normal platelet and leukocyte counts. Her thyroid tests were positive for hyperthyroidism with an undetectable TSH, T4 total 13.5 mcg/dL, T4 free 1.6 ng/dL, and T3 total 211 ng/dL. Anti-Tg and anti-TPO were negative. Thyroid iodine scan showed no radioactive iodine uptake (Figures 1(a) and 1(b)). The diagnosis of subacute thyroiditis (SAT) was confirmed.

## 2. Outcome and Follow-Up

During her follow-up visit one month after the diagnosis, the patient has remained asymptomatic, but her lab tests are still relevant for anemia, thrombocytopenia, high ESR, and low TSH (0.01 mUI/L).

## 3. Discussion

The etiology of SAT (subacute granulomatous thyroiditis) is usually viral. The most commonly associated viruses include enterovirus, coxsackievirus, mumps, measles, and adenovirus [1–3]. However, the prevalence of SAT associated with the novel coronavirus seems to be increasing as we are all more aware of its complications [4–7]. The incidence of SAT is 12.1 cases per 100,000/year, and it is more frequent in young women when compared to men (19.1 vs 4.1 per 100,000/year, respectively) [8, 9]. It is characterized by neck pain or discomfort and a tender diffuse goiter associated with myalgias, pharyngitis, low-grade fever, and fatigue [1, 10]. Up to 50% experience transient symptoms of thyrotoxicosis followed by euthyroidism, hypothyroidism, and a normalization of thyroid function within 3 months [1]. The incidence is 12.1 cases per 100,000/year with a higher incidence in young females when compared to men (19.1 vs 4.1 per 100,000/year, respectively) [8, 9]. The hallmark laboratory findings of SAT are elevated erythrocyte sedimentation rate (greater than 50 mm/hr) and C-reactive protein (CRP), low TSH concentrations with high free T4 and T3 during the early stages of the illness, and there is also a normal or mildly elevated leukocyte count and mild anemia [1]. Differently from Graves' disease, in SAT, most patients do not experience symptoms of thyroid excess, and T3 is not disproportionally elevated. With regard to antithyroid peroxidase and anti-thyroglobulin antibodies, they are usually undetectable or present at low titers. Imaging studies include a radioiodine or technetium imaging study and a Doppler ultrasound. The radioiodine study will show a low uptake (less than 1–3 percent) or a faint heterogeneous pattern of radionuclide uptake during the hyperthyroid phase. The ultrasound will show a normal or enlarged thyroid but typically, diffusely, or focally hypoechogenic, and the color Doppler sonography will demonstrate low flow.

Accepted treatment for subacute thyroiditis includes anti-inflammatory therapy with nonsteroidal anti-inflammatory drug (NSAID) or prednisone. Patients experiencing bothersome symptoms of hyperthyroidism such as palpitations, anxiety, or tremor benefit from treatment with a beta blocker such as propranolol (40–120 mg). Thionamides should not be used.

SAT is not the only thyroid condition associated with COVID-19, cases of thyroxine thyrotoxicosis have been also described by Muller et al. [6]. All available information on SAT and thyroxine thyrotoxicosis associated with COVID-19 comes from the Italian experience. There are three case reports by Ippolito et al., Brancatella et al., and Ruggeri et al. (see Table 1) and a study focusing on the prevalence of SAT and thyroxine thyrotoxicosis in patients with severe presentation of COVID-19 from an ICU unit by Muller et al. based in Milan (see Table 1) [4–7].

As shown in Table 1, similar to this Mexican case, all individual case reports of SAT associated with SARS-Cov-2 infection have been in women with an age range of 18 to 69. While all Italian cases presented symptoms of thyrotoxicosis, no such findings occurred in the Mexican case. Time between the diagnosis of SARS-Cov-2 and the onset of symptoms ranged from 5 days to one month, and the time between the diagnosis of SAT and recovery ranged from 1 week to 1 month. This differs from the findings of the study by Muller et al. which compared thyroid function tests in patients from the ICU in 2019 (without COVID-19) and patients in the ICU with COVID-19 in 2020 [6]. Findings from this study include that a higher proportion of patients from the ICU presented elevated TSH in 2020, suggesting the presence of SAT associated with SARS-Cov-2. In order to analyze if these findings were consistent with SAT, seven patients with low TSH were followed up for 55 days. While six showed laboratory findings compatible with SAT without ever developing neck pain, imaging studies compatible with SAT were found only in three of the patients. All of these findings suggest that SARS-Cov-2 thyroid infection may occur with or without clinical symptoms.

The pathophysiology of SAT associated with SARS-Cov-2 infection is thought to be similar to the association with other viral conditions, either direct infection or a postviral inflammatory reaction targeting the thyroid of genetically susceptible individuals. Muller et al. suggest that the affinity of SARS-Cov-2 to the thyroid gland is via the ACE2 receptors which are more prevalent in thyroid cells than lung cells. Because of this, clinicians should be aware of the possibility of subacute thyroiditis in patients experiencing SARS-Cov-2 infection. Another consideration is that because of the high prevalence of asymptomatic patients with SARS-Cov-2 infection, patients presenting with SAT should be tested for Sars-Cov-2. On the 21st of May, the Endocrine Society emitted a statement based on the case published by Brancatella et al.

## Figures and Tables

**Table 1:** Available case reports on SAT associated with COVID-19.

Case	Place	Gender	Age	Presentation	Laboratory findings	Imaging studies	Follow-up
Ippolito et al. [5]	Italy	Female	69	Five days after the diagnosis of COVID-19 pneumonia, she presented with palpitations, insomnia, and agitation. She did not refer neck pain but was under pain killers.	TSH: low; FT4 and FT3: high; anti-thyroglobulin antibodies (TgAb), anti-peroxidase antibodies (TPOAb), and anti-TSH receptor antibodies (TRAb): negative.	Ultrasonography (US): enlarged hypoechoic thyroid with decreased vascularity. No uptake in the thyroid scan using Tc 99-m.	She was given methimazole, but the thyrotoxicosis worsened. She was given steroids, and after 10 days, all laboratory findings and symptoms improved.
Brancatella et al. [4]	Italy	Female	18	Symptoms included fever, neck pain radiated to the jaw, and palpitations 15 days after a SARS-CoV-2-positive oropharyngeal swab.	TSH: undetectable; FT4 and FT3: elevated; TgAb, TPOAb, TRAb, inflammatory markers, and white blood cell count: elevated.	US: bilateral and diffuse hypoechoic areas.	Symptoms improved within 1 week, and thyroid function and labs normalized in 40 days.
Ruggeri et al. [7]	Italy	Female	43	The patient developed neck pain, fatigue, tremors, and palpitation one month after the diagnosis of COVID-19.	TSH: suppressed; FT4 and FT3: elevated; TgAb, TPOAb, and TRAb: undetectable.	US: diffusely enlarged and hypoechogenic thyroid gland. Thyroid scintigraphy with 99mTc-perthecnetate showed markedly reduced uptake.	The patient was treated with oral prednisone, and within 2 weeks, the inflammatory response normalized. Thyroid function tests recovered 4 weeks after steroid treatment.
Campos-Barrera et al.	Mexico	Female	37	Severe neck pain (8/10) irradiating to the right jaw and ear as well as fatigue. She did not mention any clinical signs of hyperthyroidism.	TSH: undetectable; FT4 and FT3: elevated; TgAb, TPOAb, and TRAb: undetectable; elevated ESR and CRP and anemia.	Thyroid iodine scan showed no radioactive iodine uptake.	One month after the diagnosis, the patient remained asymptomatic but her lab tests were still relevant for anemia, thrombocytopenia, high ESR, and low TSH (0.01 mUI/L).

## Data Availability

The patient's data used to support the findings of this case report are available from the corresponding author upon request.
